# Supplementary material for: Non-adherence to self-care and associated factors among diabetes adult population in Ethiopian: A systemic review with meta-analysis
Source: PLoS One. 2021 Feb 10;16(2):e0245862. doi: 10.1371/journal.pone.0245862 (PMC7875372; doi:10.1371/journal.pone.0245862)
Supplement: S1 File — (DOCX) [file pone.0245862.s001.docx]

Page 2 of 2

**Supporting information 1: Protocol and Registration**

**Protocol and Registration** is register as "**Non-Adherence to Self-Care Practice and Influencing Factors among Ethiopians Adults with Diabetes: Systemic Review with Meta-Analysis**. " which was published on a website on Apr 28, 2020. The record was published exactly as submitted with a **registration number is: CRD42020149478.** All the published document is attached in the systems as supplementary document.
