# Supplementary material for: Non-adherence to self-care and associated factors among diabetes adult population in Ethiopian: A systemic review with meta-analysis
Source: PLoS One. 2021 Feb 10;16(2):e0245862. doi: 10.1371/journal.pone.0245862 (PMC7875372; doi:10.1371/journal.pone.0245862)
Supplement: S3 File — (DOCX) [file pone.0245862.s003.docx]

**Supporting information 3:** search strategy applied to PubMed database in the current review

| Search # | Query | Items found |
| --- | --- | --- |
| #1 | **Self-care adherence OR self- care practice OR therapy adherence OR treatment adherence OR medication intake adherence OR medication compliance OR patient compliance[MeSH Terms]** | 399,284 |
| #2 | **diabetes mellitus OR diabetes[MeSH Terms]** | 506,086 |
| #3 | **Patients OR clients[MeSH Terms]** | 7,009,617 |
| #4 | **factors OR determinants OR influences OR risk factors OR predictors** | 13,838,036 |
| #5 | **Ethiopia** | 21,382 |
| #6 | **#1 AND #2 AND #3 AND #4 AND #5** | 56 |
