# Supplementary material for: Non-adherence to self-care and associated factors among diabetes adult population in Ethiopian: A systemic review with meta-analysis
Source: PLoS One. 2021 Feb 10;16(2):e0245862. doi: 10.1371/journal.pone.0245862 (PMC7875372; doi:10.1371/journal.pone.0245862)
Supplement: S4 File — (DOCX) [file pone.0245862.s004.docx]

**Supporting information 4**: Risk of bias assessment of eligible articles using the Hoy 2012 tool

| Study ID | Representation | Sampling | Random selection | Non-response bias | Data collection | Case definition | Tool Reliability and validity | Data collection method | Numerator and denominator | Summary Assessment |
| --- | --- | --- | --- | --- | --- | --- | --- | --- | --- | --- |
|  |  |  |  |  |  |  |  |  |  |  |
| Chali SW et al | Low risk | Low risk | Low risk | Low risk | Low risk | High risk | Low risk | Low risk | Low risk | Low risk |
| Dedefo MG et al | High risk | Low risk | High risk | Low risk | Low risk | Low risk | Low risk | Low risk | Low risk | Low risk |
| Ayele BH. et al | High risk | Low risk | Low risk | Low risk | Low risk | High risk | Low risk | Low risk | Low risk | Low risk |
| Gurmu Y.et al | High risk | High risk | High risk | Low risk | Low risk | High risk | Low risk | High risk | Low risk | *MR |
| Kassahun T. et al | Low risk | Low risk | High risk | Low risk | Low risk | Low risk | Low risk | Low risk | Low risk | Low risk |
| Berhe KK.et al | Low risk | High risk | Low risk | Low risk | High risk | High risk | Low risk | Low risk | High risk | *MR |
| Mamo M. et al | High risk | Low risk | High risk | Low risk | High risk | High risk | Low risk | Low risk | High risk | *MR |
| Tadesse E. et al | High risk | High risk | Low risk | Low risk | High risk | low risk | High risk | Low risk | High risk | MR |
| Tiruneh SA. et al | Low risk | Low risk | Low risk | Low risk | Low risk | Low risk | Low risk | Low risk | Low risk | Low risk |
| Ayele K. et al | High risk | High risk | Low risk | Low risk | Low risk | Low risk | Low risk | Low risk | Low risk | Low risk |
| Abate TW. et al | Low risk | Low risk | Low risk | Low risk | Low risk | Low risk | Low risk | Low risk | Low risk | Low risk |
| Niguse H. et al | High risk | Low risk | Low risk | Low risk | Low risk | Low risk | Low risk | High risk | Low risk | Low risk |
| Mariye T. et al | High risk | Low risk | High risk | Low risk | Low risk | High risk | Low risk | Low risk | Low risk | Low risk |
| Alemayhu T. et al | Low risk | Low risk | Low risk | Low risk | Low risk | High risk | Low risk | Low risk | Low risk | Low risk |
| FEYISSA L. et at | Low risk | High risk | Low risk | Low risk | Low risk | Low risk | High risk | Low risk | Low risk | Low risk |
| Berhe KK. et at | Low risk | High risk | High risk | Low risk | Low risk | High risk | Low risk | High risk | High risk | *MR |
| Addisu Y. et al | High risk | High risk | Low risk | Low risk | Low risk | Low risk | Low risk | Low risk | Low risk | Low risk |
| Sorato MM.et al | High risk | High risk | Low risk | High risk | Low risk | Low risk | Low risk | Low risk | Low risk | Low risk |
| Aschalew AY. et al | Low risk | Low risk | Low risk | Low risk | Low risk | Low risk | Low risk | Low risk | Low risk | Low risk |
| Amente T. et al | Low risk | Low risk | Low risk | Low risk | Low risk | High risk | Low risk | Low risk | Low risk | Low risk |
| Berhan T. et al | Low risk | Low risk | Low risk | Low risk | Low risk | Low risk | Low risk | Low risk | Low risk | Low risk |

*MR: moderate Risk

Risk of bias assessment tool: Yes (low risk); No (high risk)

1. Representation: Was the study target population a close representation of the national population?

2. Sampling: Was the sampling frame a true or close representation of the target population?

3. Random selection: Was some form of random selection used to select the sample OR a census?

4. Non-response bias: Was the likelihood of non-response bias minimal?

5. Data collection: Were data collected directly from the study subjects?

6. Case definition: Was an acceptable case definition used in the study?

7. Reliability and validity of study tool: Was the study instrument that measured the parameter of interest show to have reliability and validity?

8. Data collection: Was the same mode of data collection used for all subjects?

9. Numerators and denominators: Were the numerator(s) and denominator(s) for the parameter of interest appropriate?
