# Supplementary material for: Non-adherence to self-care and associated factors among diabetes adult population in Ethiopian: A systemic review with meta-analysis
Source: PLoS One. 2021 Feb 10;16(2):e0245862. doi: 10.1371/journal.pone.0245862 (PMC7875372; doi:10.1371/journal.pone.0245862)
Supplement: S5 File — (PDF) [file pone.0245862.s005.pdf]

Patient Preference and Adherence
Dovepress
open access to scientific and medical research
Open Access Full Text Article
ORIGINAL RESEARCH

Adherence to diabetic self-care practices and its associated factors among patients with type 2 diabetes in Addis Ababa, Ethiopia

This article was published in the following Dove Press journal:
Patient Preference and Adherence

Zeleke Bonger[1]
Solomon Shiferaw[2]
Eshetu Zerihun Tariku[3]

[1]TB/HIV Control and Prevention, Ethiopian Public Health Institute, Adama, Ethiopia; [2]School of Public Health, Addis Ababa University, Addis Ababa, Ethiopia; [3]Department of Public Health, Arba Minch University, Arba Minch, Ethiopia

Background: Self-care practices in diabetes patients are crucial to keep the illness under control and prevent complications. Effective management of diabetes will be a difficult task without adequate understanding of the existing level of practice related to diabetes self-care. This study is, therefore, aimed at assessing the self-care practice and its associated factors among patients with type 2 diabetes in Tikur Anbessa Specialized Hospital, Ethiopia.
Materials and methods: A health facility-based cross-sectional study was conducted among 419 type 2 diabetes patients from March 29, 2013, to May 16, 2013. The data were collected by face-to-face interview using structured and pretested questionnaire. Binary logistic regression was performed to assess the association between determinant factors and adherence to self-monitoring of blood glucose. Adjusted odds ratios (AOR) with its 95% confidence interval (CI) were estimated to identify factors associated with the outcome variables in the multivariable analysis.
Result: In this study, 318 (75.9%) diabetes patients did not adhere to the recommended diet management, 350 (83.5%) did not adhere to self-monitoring of blood glucose level, while 18 (4.3%) of the respondents did not adhere to the prescribed medications. Diabetic patients who were unemployed were 2.4 times more likely to practice blood glucose monitoring than merchants (AOR [95% CI] =2.4 [1.3–5.9]). Those who attended primary education were 70% less likely to adhere to blood glucose self-monitoring than those educated to a tertiary educational level (AOR [95% CI] =0.3 [0.1–0.9]). Respondents within the age group of 40–49 years were 11 times more likely to adhere to their medication than those aged 60–76 years (AOR [95% CI] =11 [1.03–13.6]).
Conclusion: The study showed that the extent to which individuals adhere to the recommended management of type 2 diabetes is substantially low. Improving awareness of patients and the community at large is imperative especially on medication adherence, glycemic control and diet management.
Keywords: type 2 diabetics, self-care practice, Tikur Anbessa, Ethiopia

Introduction

The World Health Organization defines diabetes as a metabolic disorder of multiple etiologies characterized by chronic hyperglycemia with disturbances of carbohydrate, fat, and protein metabolism that results from defects in insulin secretion, insulin action, or both.[1] The risk factors associated with type 2 diabetes can be grouped into 2 categories: modifiable and nonmodifiable risk factors. Modifiable risk factors include diets rich in saturated fats and simple carbohydrates, impaired glucose tolerance, metabolic syndrome, high blood pressure (≥140/90 mmHg), elevated plasma triglycerides (≥250 mg/dL), and low levels of physical activities (<3 times a week).

Correspondence: Eshetu Zerihun Tariku
College of Medicine and Health Sciences, Arba Minch University, PO Box 21, Arba Minch, Ethiopia
Tel +251 91 783 5302
Email eshetamu@gmail.com

© 2018 Bonger et al. This work is published and licensed by Dove Medical Press Limited. The full terms of this license are available at https://www.dovepress.com/terms.php and incorporate the Creative Commons Attribution – Non Commercial (unported, v3.0) License (http://creativecommons.org/licenses/by-nc/3.0/). By accessing the work you hereby accept the Terms. Non-commercial uses of the work are permitted without any further permission from Dove Medical Press Limited, provided the work is properly attributed. For permission for commercial use of this work, please see paragraphs 4.2 and 5 of our Terms (https://www.dovepress.com/terms.php).

The nonmodifiable risk factors are age (older than 45 years), family history of diabetes, ethnicity, and diabetes during a previous pregnancy.[2]

According to the International Diabetes Federation Atlas, sixth edition, approximately 382 million people had diabetes in 2013, and the number of people with the disease is set to rise beyond 592 million in $<$25 years. The majority, 80% of people, with diabetes live in low- and middle-income countries. The report also stated that Ethiopia is one of Africa's most populous countries with the highest number of people with diabetes (1.9 million).[3] Historically, diabetes was considered as a disease confined to developed countries and affluent people. However, recent estimates suggest that the prevalence of diabetes is rising globally, particularly in developing countries.[4]

Being diagnosed with a chronic illness can cause a wide range of emotional reactions, such as anger, denial, sadness, and anxiety, which can negatively affect the adjustment process. Poor patient understanding of diabetes is believed to delay appropriate self-care management, thus accelerating cardiovascular complications, stroke, and kidney failure.[5] Diabetes has an enormous impact on people, their families, the community, and the health system. It has been demonstrated that people with type 2 diabetes have significantly lower productivity and participation rates. The costs for type 2 diabetes have been rising rapidly over recent years.[6] Lack of awareness by Africans and facilities for detection and monitoring of diabetes mellitus may contribute to the high prevalence of diabetic complications.[7]

Diabetic self-care encompasses self-monitoring of blood glucose (SMBG), nutrition, physical activity, and compliance with medication. It also takes into consideration the interrelation between self-care practices and implementation of appropriate changes in the regular life cycle.[2,8,9] Glycemic control is only one among a number of appropriate self-care behaviors that are critical to good diabetes care and consequently short- and long-term outcomes. Clinical management guidelines emphasize the importance of medication adherence, physical activity, diet, and SMBG.[10]

Self-care practices in diabetes are crucial in keeping the illness under control, and as much as 95% of the self-care is usually provided by the patients or their families. Poor practices among diabetic patients are some of the important variables influencing the progression of diabetes and its complications, which are largely preventable.[11] Effective management of diabetes will be a difficult task without adequate understanding of the existing level of practice related to diabetes self-care. The status of patient self-care practice and factors influencing self-care practice of type 2 diabetic patients was not well known in Ethiopia. This study is, therefore, aimed at assessing the self-care practice and its associated factors among type 2 diabetic patients in Tikur Anbessa Specialized Hospital, Addis Ababa, Lideta Sub City, Ethiopia. The finding of this study would be helpful for health professionals to design appropriate intervention strategies to improve adherence to diabetic self-care practices among diabetic patients.

Materials and methods

Setting and design

A health-facility-based cross-sectional study design was employed in the endocrinology unit of Tikur Anbessa Specialized Hospital between March 29, 2013, and May 16, 2013.

Study participants

Four hundred twenty-two type 2 diabetes patients who were older than 18 years and visited the endocrinology unit of Tikur Anbessa Specialized Hospital diabetes center for follow-up during data collection period were included in the study. Patients who were seriously ill during the time of data collection (since they would not able to respond to the questions) and newly diagnosed type 2 patients (those who have $<$1-year follow-up period) were excluded from the study. Every consecutive patient who met the inclusion criteria were selected until the required sample size was achieved.

Data collection instruments

The data were collected by face-to-face interview using structured and pretested questionnaire. The questionnaire was developed on the basis of various prior similar studies and further modified to include important variables of this study. The questionnaire included questions that were used to assess diabetic risk factors, demographic and economic characteristics, duration of diabetes, lifestyle, and health service-related factors. The questionnaire was initially prepared in English and translated to Amharic, the local language, and again backtranslated to English by independent language experts. The data were collected by 6 trained nurses with diplomas and 1 supervisor.

Data quality assurance

The quality of data was assured by properly designing the tool, and the questionnaire was pretested in St Pauls Generalized Specialized Hospital using 5% of the sample size, and important modifications were made prior to the actual data collection. Two days of intensive training was given to the data collectors and supervisor to help them familiarize themselves with the tool, method of data collection, how to

ask questions, the way of approaching respondents, and how to rate responses. The collected data were checked carefully on a daily basis for completeness, accuracy, and clarity by a supervisor, and the principal investigators monitored the overall activities of data collection.

Data processing and analysis

The data were coded and entered into EPI-INFO version 3.5.3 (Centers for Disease Control and Prevention, Atlanta, GA, USA) and exported to SPSS version 16 (SPSS Inc., Chicago, IL, USA) for further analysis. Descriptive statistics were used to describe the data. Binary logistic regression was performed to assess the association between determinant factors and adherence to self-monitoring of blood glucose. All the covariates that were significant at $p<0.2$ in the bivariate analysis were considered for further multivariate analysis. Using a multivariable logistic regression, odds ratios (ORs), along with 95% confidence interval (CI), were estimated to identify the factors associated with the outcome variables. The level of statistical significance was declared at $p<0.05$. The model was tested using the Hosmer–Lemeshow goodness of fit test.

Operational definitions

Practice of self-care: The correct answer for any practice items was given a score of "1," and the incorrect given "0." The scores of each area of practice were added and converted into a percent score. The total score, the level of practice, was classified into two: <59% and below (unsatisfactory practice of diabetic self-care) and 60% and above (satisfactory practice).[12,13]

Adherence to physical activity: At least 30–60 minutes of moderate aerobic activity per day or ≥3 days per week.[14]

Ethical considerations

Prior to actual data collection, ethical clearance was obtained from Addis Ababa University, School of Public Health, Research, and Ethical Review Committee, with protocol number SPH/031/05. Written informed consent was obtained from each study participant. The respondents were assured of the confidentiality by excluding their names during the data collection. They were well informed that they had full right to refuse to participate and/or withdraw from the study at any time without any precondition or consequences.

Results

Sociodemographic and economic characteristics

From the total of 422 diabetic patients who consented to participate in the study, only 3 participants were excluded because of incomplete responses (making the response rate at 99.3%). Out of the total participants, 237 (56.6%) were female and 182 (43.4%) were male. A similar percentages 123 (29.4%) of respondents were in the age range of 40–49 and 50–59 years. One hundred nine (26%) and 64 (15.2%) of respondents were between the ages of 60–76 and 30–39 years, respectively. The mean age ± SD for the studied participants was 51.1±10.6 years. The majority, 310 (73.7%), of the respondents were married. Concerning educational status, 120 (28.6%), 138 (32.9%), and 113 (27%) of the participants had tertiary, secondary, and primary education, respectively, whereas 48 (11.5%) of the respondents were unable to read and write. Approximately, half of the participants (53.3%) had a 2–9 years follow-up period in the endocrinology unit of Tikur Anbessa Specialized Hospital (Table 1).

Adherence to diabetic self-care practices

Adherence to exercise

The results of exercise showed that 194 (46.3%) of the study subjects adhered to physical exercise, which means they were performed at least 30–60 minutes of moderate aerobic activity per day or ≥3 days per week. Majority of respondents, 104 (53.6%), were physically exercising for 30–45 minutes per session (Figure 1).

Adherence to prescribed medications

A total of 401 (95.7%) of respondents were reported that they adhered to their medications, whereas only 18 (4.3%) of the respondents did not adhere to the prescribed antidiabetic medications. Majority of the study participants, 244 (58%), were taking insulin, 151 (36%) of them were taking oral hypoglycemic agents, and 24 (6%) of participants taking both.

Adherence to dietary management

The majority, 318 (75.9%), of the study participants did not adhere to recommended dietary management practices.

Adherence to SMBG

The majority, 350 (83.5%), of the study participants did not adhere to SMBG, which means they monitored their blood glucose levels <1–2 times per week; only 69 (16.5%) patients adhered, meaning they monitored their blood glucose at least 3–4 times a week. Among respondents who were adhered to SMBG, 33 (7.9%) and 36 (8.6%) were male and female, respectively, and out of all respondents who did not adhere, 149 (35.6%) and 201 (48%) were male and female, respectively.

Table 1 Sociodemographic and economic characteristics of the study participants (n=419)

Variables	Category	Frequency	Percent
Sex	Male	182	43.4
	Female	237	56.6
Religion	Ethiopian orthodox	273	65.2
	Muslim	62	14.8
	Protestant	84	20
Age (years)	30–39	64	15.2
	40–49	123	29.4
	50–59	123	29.4
	60–76	109	26
Educational status	Unable to read and write	48	11.5
	Primary level (1–8 grade)	113	27
	Secondary level (9–10/12 grade)	138	32.9
	Tertiary level (>12 grade)	120	28.6
Marital status	Married	310	74
	Single	58	13.8
	Divorced	23	5.5
	Widowed	28	6.7
Occupational status	Employed	122	29.1
	Unemployed	179	42.7
	Merchant	118	28.2
Place of residence	Urban	399	95.2
	Rural	20	4.8
Average monthly income (Birr)	446–1,200	223	53.3
	1,201–2,500	148	35.3
	2,501–9,000	48	11.4
Number of years of follow-up	<2	64	15.3
	2–9	260	62
	10–24	95	22.7

Factors affecting adherence to diabetic self-care practices

In the multivariable logistic regression analysis, level of education and occupational status of study subjects was found to have a statistically significant association with adherence to SMBG practice. Patients who are unemployed were about 2.4 times more likely to have better adherence to SMBG practice than merchants (adjusted OR [AOR] [95% CI] =2.4 [1.3–5.9]). Respondents who had primary educational level were 70% less to adhere to SMBG than those who had a tertiary educational level (Table 2). Respondents within the age group of 40–49 years were 11 times more likely to be adhered to their prescribed antidiabetic medications compared with those aged 60–76 years, (AOR [95% CI] =11 [1.03–13.6]) (Table 3).

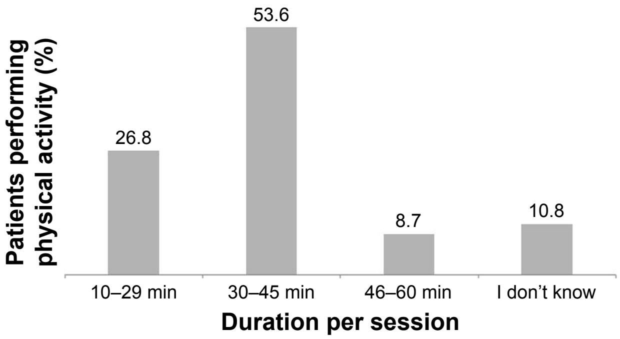

Figure 1 Duration of exercise in type 2 diabetes patients (n=419).

Discussion

The extent to which individuals (diabetic patients) are able to adhere to diabetic self-care recommendations varies. Despite the increasing prevalence of diabetes, improved understanding of the disease and glycemic control does not appear to be improving in Ethiopia.

A regular program of physical activity helps to reduce the body weight and decrease glucose intolerance and the occurrence of complications. In this study, 194 (46.3%) of the study subjects adhered to physical exercise activity. Even though the finding is higher than the findings from a study in Yemen, where only 15.2% of participants reported good adherence to the recommended levels of physical exercise,[15] and hence, diabetic patients should be encouraged to develop the habit of performing physical exercises. The discrepancy might be attributed to the difference in

Table 2 Association between covariates and adherence to SMBG (n=419)

Variable	Adherence to blood glucose monitoring, n (%)		COR (95% CI)	AOR (95% CI)
	Adhered	Not adhered		
Sex				
Male	33 (7.9)	149 (35.6)	1.237 (0.74–2.07)	1.1 (0.6–2.3)
Female	36 (8.6)	201 (48)		1.00
Religion				
Orthodox	34 (8)	240 (57.3)	1.3 (0.6–2.9)	1.2 (0.5–2.9)
Muslim	6 (1.4)	56 (13.4)	0.9 (0.3–2.8)	0.8 (0.3–2.8)
Protestant	8 (4.3)	75 (18)	1.0	1.0
Age (years)				
30–39	13 (3.1)	51 (12.2)	1.1 (0.5–2.2)	1.1 (0.3–3.4)
40–49	16 (3.8)	107 (25.5)	0.6 (0.3–1.2)	0.6 (0.2–1.8)
50–59	18 (4.3)	105 (25)	0.7 (0.4–1.3)	0.6 (0.2–1.4)
60–76	22 (5.3)	87 (20.8)	1.0	1.0
Marital status				
Married	53 (12.6)	257 (61.3)	1.237 (0.412–3.714)	1.1 (0.3–4.8)
Single	10 (2.4)	48 (11.5)	1.25 (0.35–4.40)	0.5 (0.1–3.2)
Divorced	2 (0.5)	21 (5)	0.6 (0.1–3.4)	0.7 (0.1–6.5)
Widowed	4 (1)	24 (5.7)	1.0	1.0
Educational status				
Unable to read and write	7 (1.7)	41 (9.9)	0.54 (0.22–1.32)	0.4 (0.1–1.5)
Primary level	13 (3.1)	100 (23.9)	0.4 (0.2–0.8)*	0.3 (0.1–0.9)*
Secondary level	20 (4.8)	118 (28.2)	0.5 (0.3–1.0)	0.8 (0.3–2.1)
Tertiary level	29 (6.9)	91 (21.7)	1.0	1.0
Average monthly income (Birr)				
446–1,200	34 (8.2)	184 (45.1)	0.6 (0.2–1.9)	0.6 (0.1–3.5)
1,201–2,500	28 (6.9)	120 (28.6)	0.7 (0.2–1.9)	0.8 (0.1–3.9)
2,501–9,000	7 (1.7)	41 (9.8)	1.0	1.0
Residence				
Urban	66 (15.8)	333 (79.4)	1.1 (0.3–3.9)	0.5 (0.1–2.3)
Rural	3 (0.7)	17 (4)	1.0	1.0
Occupational status				
Employed	23 (5.5)	96 (23)	1.5 (0.8–2.8)	1.1 (0.5–2.9)
Unemployed	22 (5.3)	155 (37)	1.5 (0.8–2.8)*	2.4 (1.3–5.9)*
Merchant	24 (5.7)	99 (23.6)	1.0	1.0
Duration of service following (years)				
<2	7 (1.7)	57 (13.6)	0.5 (0.2–1.3)	0.76 (0.2–2.6)
2–9	44 (10.5)	216 (51.5)	0.9 (0.5–1.6)	0.9 (0.4–2.1)
10–24	18 (4.3)	77 (18.4)	1.0	1.0

Note: *Statistically significant ($p<0.05$).
Abbreviations: AOR, adjusted odds ratio; CI, confidence interval; COR, crude odds ratio; SMBG, self-monitoring of blood glucose.

lifestyle (most of the diabetes patients in Ethiopia do not lead a sedentary life, they perform physical exercise daily, at least a simple walk for half an hour each day), and most of the respondents in this study were relatively not very old. On the contrary, the level of adherence to physical activity reported in the present study is lower than a finding from a study in Mekelle, Ethiopia, where 74.0% diabetic patients adhered to physical activities that met the recommended guidelines.[16] The self-reported nature of the data collection method used for the assessment of adherence to exercise might have contributed to such a variation.

The present study showed that only 69 (16.5%) of respondents adhere to SMBG practices. This conforms to a report from the study in Mekelle, Ethiopia, and Ghana, where the majority (86.0%) of the study participants did not adhere to SMBG.[16,17] The low adherence to SMBG practices could probably be attributed to relevant financial barriers to purchase glucometer or undergo a check-up at a health facility. In this study, only 4.3% of respondents did not adhere to prescribed medications. This finding is lower when compared to similar researches done in Jimma, Ethiopia, where 23.6% and 24.9% of patients poorly adhered to the prescribed medication.[18,19] It is also lower when compared with a result

Table 3 Association between covariates and adherence to prescribed medications (n=419)

Variable	Medication adherence, n (%)		COR (95% CI)	AOR (95% CI)
	Adhered	Not adhered		
Sex				
Male	174 (41)	8 (1.9)	0.9 (0.3–2.5)	0.7 (0.2–2.2)
Female	227 (54.2)	10 (2.4)	1.0	1.0
Religion				
Orthodox	261 (62.3)	13 (3.1)	0.5 (0.1–2.2)	1.2 (0.5–3)
Muslim	59 (14)	3 (0.7)	0.5 (0.1–3)	0.8 (0.3–2.8)
Protestant	81 (19.3)	2 (0.4)	1.0	1.0
Age (years)				
30–39	60 (14.3)	44 (10.5)	0.9 (0.3–3.2)	0.7 (0.1–4.0)
40–49	122 (29.1)	1 (0.2)	7.1 (0.8–5.9)*	11 (1.0–13.6)*
50–59	116 (27.7)	7 (1.7)	0.9 (0.3–2.9)	1.6 (0.4–6.1)
60–76	103 (24.6)	6 (1.4)	1.0	1.0
Marital status				
Married	296 (70.6)	14 (3.3)	0.8 (0.1–6.2)	0.5 (0.1–6.4)
Single	57 (13.6)	1 (0.2)	2.1 (0.1–35)	5.4 (0.1–212.9)
Divorced	21 (5)	2 (0.5)	0.4 (0.1–4.6)	0.2 (0.01–4.8)
Widowed	27 (6.4)	1 (0.2)	1.0	1.0
Educational status				
Unable to read and write	44 (10.5)	4 (1)	0.5 (0.12–1.9)	0.4 (0.1–2.4)
Primary level	110 (26.3)	3 (0.7)	1.6 (0.4–6.8)	4.8 (0.8–29)
Secondary level	132 (31.5)	6 (1.4)	0.9 (0.3–3.2)	1.3 (0.4–5.6)
Tertiary level	115 (27.4)	5 (1.2)	1.0	1.0
Average monthly income (Birr)				
446–1,200	215 (50.5)	10 (2.4)	1.1 (0.4–2.5)	0.9 (0.3–2.7)
1,201–2,500	140 (33.4)	8 (1.9)	1.4 (0.6–3.4)	1.5 (0.6–4.2)
2,501–9,000	48 (11.4)	–	1.0	1.0
Residence				
Urban	383 (91.4)	16 (3.8)	2.7 (0.6–12.5)	3.3 (0.5–20)
Rural	18 (4.3)	2 (0.5)	1.0	1.0
Occupational status				
Employed	117 (27.9)	5 (1.2)	0.9 (0.2–2.7)	0.9 (0.2–3.7)
Unemployed	111 (26.5)	7 (1.7)	0.6 (0.2–1.7)	0.5 (0.1–1.9)
Merchant	173 (41.3)	6 (1.4)	1.0	1.0
Duration of service follow up (years)				
<2	59 (14)	5 (1.2)	0.5 (0.1–2)	0.4 (0.1–2.2)
2–9	251 (59.9)	9 (2.1)	1.2 (0.4–4.1)	1.7 (0.4–7.4)
10–24	91 (21.7)	4 (1)	1.0	1.0
Medication type				
Oral hypoglycemic	141 (33.7)	10 (2.4)	0.6 (0.1–4.5)	0.4 (0.1–5.5)
Insulin	237 (56.6)	7 (1.7)	1.5 (0.1–18.9)	1.7 (0.1–23)
Both	23 (5.4)	1 (0.2)	1.0	1.0

Note: *Statistically significant ($p<0.05$).
Abbreviations: AOR, adjusted odds ratio; CI, confidence interval; COR, crude odds ratio.

from the study conducted in Assela (31.2%) and Gondar, Ethiopia (14.9%).[20,21] Increased the level of adherence to prescribed medication in this study might be attributed to the relatively better educational status of the study populations in the study area and the socioeconomic difference among the study areas.

In this study, only 101 (24%) participants adhered to diet management practice. This finding is lower than the figures reported from studies in Egypt (37%) and India (45. 9%).[13,22] This low result might be due to the inability to distinguish clearly between low- and high-carbohydrate index food items and due to eating outside the house. In addition, the socio-economic difference among the study subjects may, in turn, have contributed to the difference in food preference capacity. The level of adherence to diet management observed in this study is higher when compared with a research study

from Ghana, where only 8.6% of studied patients followed the recommended diet.[17] This variation might be attributed to the difference in sample size and the study period. Among patients who adhered to diet management practices, 21 (5%) subjects stated honey and sugar are food items that should be avoided to manage diabetes and 80 (19.1%) subjects stated that honey, marmarata, and cake are food items that should be avoided to manage diabetes. A similar research done in Nigeria showed that 53.1% of respondents mentioned excessive and prolonged intake of carbohydrate items with high glycemic index are food items that should be avoided to manage diabetes.[23,24]

The result of this study indicated that there was a significant association between medication adherence and younger age (age group 40–49 years). Similarly, a result from the study done in Nigeria and Yemen showed that the younger patients were most likely to retain what they were thought and that they remembered faster and had better recall abilities than older patients.[1,25] As indicated in this study, participants who adhered to medication were higher than the those did not; only 18 (4.3%) of the study subjects did not adhere. This might be because of the participants being well informed and because they have a good perception of the prescribed medication than other recommendations.

The present study affirmed that a statistically significant association was found between the covariate educational level and adherence to SMBG practice. Diabetic patients who were educated to a primary level were 70% less likely to adhere to SMBG than those who had a tertiary education. This is supported by findings from the studies in Jimma, Assela, and Gondar Ethiopia where patients with educational status secondary and above were more likely to attain adequate glycemic control compared to illiterate.[18,20,21] This could be attributed to the fact that the understanding and knowledge of self-care practice increased with an increased level of education. In this study, unemployed patients were found to better adhere to SMBG practice than merchants; this is supported by evidence from a study done in Jimma where being a merchant was associated with low adherence to medications.[19] This might be related to the habit of not spending sufficient time to care for themselves, a finding that has been observed among the majority of merchants in Ethiopia.

The study enrolled the patients only from 1 hospital and hence cannot be generalized to the diabetic population of Ethiopia. The study is limited by selection bias since only those patients who have visited the health facilities for interventions were included. The possibility of introducing social desirability bias is another limitation of this study since a self-reported method of data collection was used. Moreover, the study is cross-sectional in nature, and hence, the causal relationship between the covariates and outcome variables cannot be established.

Conclusion

The study showed that the extent to which individuals adhere to the recommended management of type 2 diabetes is substantially low. Improving awareness of patients and the community at large is imperative especially on medication adherence, glycemic control and diet management. Age, level of education, and occupational status were found to have a statistically significant association with adherence to self-care practice.

Finally, we would like to recommend that the diabetic center of Tikur Anbessa Specialized Hospital do more in order to improve patient's adherence to SMBG using model service users and strong service link education. Patients need to have regular blood glucose checking done at home using glucometers, and they must also be taught the importance of to performing a moderate level of exercise to manage their diabetes.

Acknowledgments

We would like to thank Doctor Tedla Kebede who greatly helped us in the recruitment of study participants. We are also thankful for the financial support provided by the Oromia Regional Health Bureau and Addis Ababa University, School of Public Health.

Author contributions

All authors contributed toward data analysis, drafting and critically revising the paper and agree to be accountable for all aspects of the work.

Disclosure

The authors report no conflicts of interest in this work.

References

1. Badran M, Laher I. Type II diabetes mellitus in Arabic-speaking countries. *Int J Endocrinol*. 2012;2012:902873.
2. Castro FG, Shaibi GQ, Boehm-Smith E. Ecodevelopmental contexts for preventing type 2 diabetes in Latino and other racial/ethnic minority populations. *J Behav Med*. 2009;32(1):89–105.
3. Joshi SR. *The IDF Diabetes Atlas*. 6th ed. Brussels, Belgium: International Diabetes Federation; 2013.
4. Rabkin M, Melaku Z, Bruce K, et al. Strengthening health systems for chronic care: leveraging HIV programs to support diabetes services in Ethiopia and Swaziland. *J Trop Med*. 2012;2012:137460.
5. Sicree R, Shaw J, Zimmet P. Diabetes and Impaired Glucose Tolerance. In: *International Diabetes Federation Diabetes Atlas*. 4th ed. Brussels: International Diabetes Federation; 2010.

6. Kheir N, Greer W, Yousif A, Al Geed H, Al Okkah R. Knowledge, attitude, and practices of Qatari patients with type 2 diabetes mellitus. *Int J Pharm Pract*. 2010;19(3):185–191.
7. Padma K, Bele SD, Bodhare TN, Valsangkar S. Evaluation of knowledge and self-care practices in diabetic patients and their role in disease management. *Natl J Res Community Med*. 2011;3(1):3–6.
8. McGill M. *Diabetes Education Training Manual for Sub-Saharan Africa*. Cape Town, South Africa: International Diabetes Federation Africa Region; 2006.
9. Kiberenge MW, Ndegwa ZM, Njenga EW, Muchem EW. Knowledge, attitude and practices related to diabetes among community members in four provinces in Kenya. *Pan Afr Med J*. 2010;7:2.
10. Laverick J. Diabetes self-management guidelines for providing services to people newly diagnosed with type 2 diabetes. Victorian Government Department of Human Services; 2007.
11. Hailu E, Mariam WH, Belachew T, Birhanu Z. Self-care practice and glycaemic control amongst adults with diabetes at the Jimma University Specialized Hospital in south-west Ethiopia. *Afr J Prim Health Care Fam Med*. 2012;4(1):311.
12. Shah VN, Kamdar PK, Shah N. Assessing the knowledge, attitudes and practice of type 2 diabetes among patients of Saurashtra region, Ethiopia. *Int J Diabetes Dev Ctries*. 2009;29(3):118–122.
13. Ali ZH. Health, and knowledge progress among diabetic patients after implementation of a Nursing Care Program based on their profile. *J Diabetes Metab*. 2010;2:121.
14. Harris P, Mann L. *Diabetes Management in General Practice*. Turner, Australia: Diabetes Australia; 2010.
15. ALhariri A, Daud F, Almaiman A, Saghir SAM. Factors associated with adherence to diet and exercise among type 2 diabetes patients in Hodeidah city, Yemen. *Diabetes Manag*. 2016;7(3):264–271.
16. Berhe KK, Gebru HB, Kahsay HB, Kahsay AA. Assessment of self care management and its associated factors among type 2 diabetes patients in Mekelle Hospital and Ayder Referral Hospitals, Mekelle City, Tigray, Northern Ethiopia, 2012/13. *Global J Med Res: F Diseases*. 2017;17(1):31–48.
17. Mogre V, Abanga ZO, Tzelepis F, Johnson NA, Paul C. Adherence to and factors associated with self-care behaviours in type 2 diabetes patients in Ghana. *BMC Endocr Disord*. 2017;17(1):20.
18. Bayisa B, Bekele M. Glycemic control and associated factors among type II diabetic patients on chronic follow up at southwest Ethiopia. *J Med Health Sci*. 2017;6(3).
19. Kassahun T, Gesesew H, Mwanri L, Eshetie T. Diabetes related knowledge, self-care behaviors and adherence to medications among diabetic patients in Southwest Ethiopia: a cross-sectional survey. *BMC Endocr Disord*. 2016;16(1):28.
20. Kassahun A, Gashe F, Mulisa E, Rike WA. Nonadherence and factors affecting adherence of diabetic patients to anti-diabetic medication in Assela General Hospital, Oromia Region, Ethiopia. *J Pharm Bioallied Sci*. 2016;8(2):124–129.
21. Abebaw M, Messele A, Hailu M, Zewdu F. Adherence and associated factors towards antidiabetic medication among type II diabetic patients on follow-up at University of Gondar Hospital, Northwest Ethiopia. *Adv Nurs*. 2016;2016:1–7.
22. Rajasekharan D, Kulkarni V, Unnikrishnan B, Kumar N, Holla R, Thapar R. Self-care actvites among patients with diabetes attending a tertiary care hospital in Mangalore Karnataka, India. *Ann Med Health Sci Res*. 2015;5(1):59–64.
23. Adisa R, Fakeye TO, Okorie LK. Knowledge, attitude and self-management practices of patients with type 2 diabetes in an ambulatory care setting in Ibadan, Nigeria. *Ethiop Pharmaceut J*. 2010;28(2).
24. Uchenna OV, Ijeoma EO, Peace IN, Ngozi KI. Knowledge of diabetes management and control by diabetic patients at Federal Medical Center Umuahia. *Int J Med and Med Sci*. 2009;1(9):353–358.
25. Adibe Maxwell O, Aguwa Cletus N, Ukwe Chinwe V, Okonta Jegberime M, Udeogaranya Obinna P. Diabetes self-care knowledge among type 2 diabetic outpatients in south-eastern Nigeria. *Int J Drug Develop Res*. 2009;1(1):85–104.

Patient Preference and Adherence Dovepress

Publish your work in this journal

Patient Preference and Adherence is an international, peer-reviewed, open access journal that focuses on the growing importance of patient preference and adherence throughout the therapeutic continuum. Patient satisfaction, acceptability, quality of life, compliance, persistence and their role in developing new therapeutic modalities and compounds to optimize clinical outcomes for existing disease states are major areas of interest for the journal. This journal has been accepted for indexing on PubMed Central. The manuscript management system is completely online and includes a very quick and fair peer-review system, which is all easy to use. Visit http://www.dovepress.com/testimonials.php to read real quotes from published authors.

Submit your manuscript here: http://www.dovepress.com/patient-preference-and-adherence-journal
