# Supplementary material for: Non-adherence to self-care and associated factors among diabetes adult population in Ethiopian: A systemic review with meta-analysis
Source: PLoS One. 2021 Feb 10;16(2):e0245862. doi: 10.1371/journal.pone.0245862 (PMC7875372; doi:10.1371/journal.pone.0245862)
Supplement: S6 File — (PDF) [file pone.0245862.s006.pdf]

National Institute for Health Research

University of York
Centre for Reviews and Dissemination

Systematic review

1. * Review title.

Give the working title of the review, for example the one used for obtaining funding. Ideally the title should state succinctly the interventions or exposures being reviewed and the associated health or social problems. Where appropriate, the title should use the PI(E)COS structure to contain information on the Participants, Intervention (or Exposure) and Comparison groups, the Outcomes to be measured and Study designs to be included.

Non-Adherence to Self-Care Practice and Influencing Factors among Ethiopians Adults with Diabetes:

Systemic Review with Meta-Analysis.

2. Original language title.

For reviews in languages other than English, this field should be used to enter the title in the language of the review. This will be displayed together with the English language title.

3. * Anticipated or actual start date.

Give the date when the systematic review commenced, or is expected to commence.

03/09/2019

4. * Anticipated completion date.

Give the date by which the review is expected to be completed.

29/02/2020

5. * Stage of review at time of this submission.

Indicate the stage of progress of the review by ticking the relevant Started and Completed boxes. Additional information may be added in the free text box provided.
Please note: Reviews that have progressed beyond the point of completing data extraction at the time of initial registration are not eligible for inclusion in PROSPERO. Should evidence of incorrect status and/or completion date being supplied at the time of submission come to light, the content of the PROSPERO record will be removed leaving only the title and named contact details and a statement that inaccuracies in the stage of the review date had been identified.
This field should be updated when any amendments are made to a published record and on completion and publication of the review. If this field was pre-populated from the initial screening questions then you are not able to edit it until the record is published.

The review has not yet started: Yes

Review stage	Started	Completed
Preliminary searches	No	No
Piloting of the study selection process	No	No
Formal screening of search results against eligibility criteria	No	No
Data extraction	No	No
Risk of bias (quality) assessment	No	No
Data analysis	No	No

Provide any other relevant information about the stage of the review here (e.g. Funded proposal, protocol not yet finalised).

6. * Named contact.

The named contact acts as the guarantor for the accuracy of the information presented in the register record.

Teshager Abate

Email salutation (e.g. "Dr Smith" or "Joanne") for correspondence:

Mr Abate

7. * Named contact email.

Give the electronic mail address of the named contact.

teshagerhylemarriam@gmail.com

8. Named contact address

Give the full postal address for the named contact.

9. Named contact phone number.

Give the telephone number for the named contact, including international dialling code.

+251912886162

10. * Organisational affiliation of the review.

Full title of the organisational affiliations for this review and website address if available. This field may be completed as 'None' if the review is not affiliated to any organisation.

Bahir Dar University

Organisation web address:

11. * Review team members and their organisational affiliations.

Give the personal details and the organisational affiliations of each member of the review team. Affiliation refers to groups or organisations to which review team members belong. **NOTE: email and country are now mandatory fields for each person.**

Mr Teshager Abate. Bahir Dar University
Mr Getenet Dessie. Bahir Dar University
Mr Emiru Ayalew. Bahir Dar University
Assistant/Associate Professor Haileyesus gedamu. Bahir Dar University

12. * Funding sources/sponsors.

Give details of the individuals, organizations, groups or other legal entities who take responsibility for initiating, managing, sponsoring and/or financing the review. Include any unique identification numbers assigned to the review by the individuals or bodies listed.

Not applicable

Grant number(s)

13. * Conflicts of interest.

List any conditions that could lead to actual or perceived undue influence on judgements concerning the main topic investigated in the review.

None

14. Collaborators.

Give the name and affiliation of any individuals or organisations who are working on the review but who are not listed as review team members. **NOTE: email and country are now mandatory fields for each person.**

15. * Review question.

State the question(s) to be addressed by the review, clearly and precisely. Review questions may be specific or broad. It may be appropriate to break very broad questions down into a series of related more specific questions. Questions may be framed or refined using PI(E)COS where relevant.

What is the adherence level of self-care practices among diabetes type 2 patients in Sub-Saharan Africa?What are factors affecting adherence of self-care practices among diabetes type 2 patients in Sub-Saharan Africa?

16. * Searches.

State the sources that will be searched. Give the search dates, and any restrictions (e.g. language or publication period). Do NOT enter the full search strategy (it may be provided as a link or attachment.)

The search strategy will be both published and unpublished studies. PubMed, Google Scholar, text words contained in the title and abstract, and of the index terms used to describe the article will be undertaken. The databases to be searched include: PubMed, HINARI, Google Scholar, Hinary and Google. The search for unpublished studies will include: Thesis, and dissertations.

The study will be included: all English-language, full-text articles for studies conducted in Sun-Saharan Africa

17. URL to search strategy.

Give a link to a published pdf/word document detailing either the search strategy or an example of a search strategy for a specific database if available (including the keywords that will be used in the search strategies), or upload your search strategy.Do NOT provide links to your search results.

Alternatively, upload your search strategy to CRD in pdf format. Please note that by doing so you are consenting to the file being made publicly accessible.

Do not make this file publicly available until the review is complete

18. * Condition or domain being studied.

Give a short description of the disease, condition or healthcare domain being studied. This could include health and wellbeing outcomes.

The study will include self reported self-care practice studies conducted among DM type 2 patients in Sub-Saharan Africa.

19. * Participants/population.

Give summary criteria for the participants or populations being studied by the review. The preferred format includes details of both inclusion and exclusion criteria.

This review will consider studies reporting self-Care practice of people with type 2 diabetes mellitus. If the study include both type2 and type 2 DM, it will be included if self-care practices for patients with type 2 diabetes presented separately only; if not it will be not considered

20. * Intervention(s), exposure(s).

Give full and clear descriptions or definitions of the nature of the interventions or the exposures to be reviewed.

The intervention group were participants who had good self-care practice

21. * Comparator(s)/control.

Where relevant, give details of the alternatives against which the main subject/topic of the review will be compared (e.g. another intervention or a non-exposed control group). The preferred format includes details of both inclusion and exclusion criteria.

The comparator group were participants who had unfavorable self-care practice

22. * Types of study to be included.

Give details of the types of study (study designs) eligible for inclusion in the review. If there are no restrictions on the types of study design eligible for inclusion, or certain study types are excluded, this should be stated. The preferred format includes details of both inclusion and exclusion criteria.

The Preferred Reporting Items for Systematic Reviews and Meta-Analyses guidelines will be employed to plan . A systematic literature search will be conducted in PubMed, Embase, Google Scholar, CINAHL, MEDLINE, and Hinari. These search terms will be pre-defined to allow a comprehensive search strategy and Medical Subject Headings (MeSH terms). we will also be used the Boolean operator in our search as they outline in their paper.The eligible criteria of this review will be used: research intended to examine the association between diabetes self- care practice and related factors, conducted Ethiopian adult diabetes patient aged 15 and above, articles published in English language, and all cross-sectional in self-care practice in peer-reviewed journals or found in the grey literature will be eligible for inclusion. Case series, case reports, cohort studies; and studies that did not report specific outcomes for self-care adherence/non-

adherence quantitatively will be excluded.

23. Context.

Give summary details of the setting and other relevant characteristics which help define the inclusion or exclusion criteria.

24. * Main outcome(s).

Give the pre-specified main (most important) outcomes of the review, including details of how the outcome is defined and measured and when these measurement are made, if these are part of the review inclusion criteria.

The main outcome of our study will be adherence level of self-care practice as defined as summary of diabetes self-care activities (SDSCA)

* Measures of effect

Please specify the effect measure(s) for you main outcome(s) e.g. relative risks, odds ratios, risk difference, and/or 'number needed to treat.

The main aim of this review will be to determine the pooled prevalence of non-adherence to self-care practice among adult diabetes patients in Ethiopia. The prevalence will be measured as the number of adult diabetes with non-adherence to self-care practice in the studies divided by the total number of diabetes patients in a study multiplied by 100. For analysis of the secondary outcomes (factors associated with non-adherence to self-care practice), we will be extracted data on factors that had been found to be related to non- adherence to self-care practice in the literature, such as the age, sex, occupation, marital status social support, owing private glucometer, duration of diabetes, diabetes-related complication, diabetes knowledge, participate diabetes education, self-efficacy, perceive severity and barrier residency and educational status. The other criterion for selecting variables was how frequently they were reported in studies included in the meta-analysis. In examining factors associated with self-care non-adherence, data will be used from the primary studies of the adjusted odd ratio (AOR) to determine the association between each of the independent variables and having non-adherence to self-care practice.

25. * Additional outcome(s).

List the pre-specified additional outcomes of the review, with a similar level of detail to that required for main outcomes. Where there are no additional outcomes please state 'None' or 'Not applicable' as appropriate to the review

Not applicable

* Measures of effect

Please specify the effect measure(s) for you additional outcome(s) e.g. relative risks, odds ratios, risk difference, and/or 'number needed to treat.

Not applicable

26. * Data extraction (selection and coding).

Describe how studies will be selected for inclusion. State what data will be extracted or obtained. State how this will be done and recorded.

Firstly, different websites and data sources will be explored to check the availability of similar studies and to avoid duplications. Then, to gather published primary studies, databases such as PubMed/MEDLINE, Scopus, and the Health Internetwork Access to Research Initiative (HINARI) will be used, and extra articles and grey literature will be also retrieved using electronics search engines of Google and Google Scholar. Also, the reference lists of the relevant articles will be tracked to access extra studies. To suppress the number of irrelevant articles during the searching process, the searching will be limited to "human studies", published in English language and studies conducted period. The search strategy will be built using keywords that have been identified using the "Medical Subject Headings (MeSH)" terms and "All fields" by linking "AND "and "OR" Boolean operator terms as appropriate, yielding a search strategy of (Diabet*) OR Diabet*[MeSH Terms] AND self care OR self-care OR self-care management OR management OR compliance AND practice* AND determin* OR associate* OR predictor* AND Ethiopia. Lastly,

the selection process will be conducted in two stages: an initial screening of titles and abstracts against the predetermined inclusion/exclusion criteria, followed by a second screening of the full text of the research reports identified as probably relevant in the initial screening. Both stages will be carried out independently by two authors, and disagreements resolved by discussion with other authors determined final eligibility of the included articles. Articles that fulfilled the prior criteria will be used as source of data for the final analysis.

27. * Risk of bias (quality) assessment.

Describe the method of assessing risk of bias or quality assessment. State which characteristics of the studies will be assessed and any formal risk of bias tools that will be used.

The database search results will be exported and duplicate articles will be removed using EndNote software (version X7; Thomson Reuters, New York, NY). The New castle Ottawa Scale (NOS) criteria will be used for quality assessment of selected studies before analysis. Two independent reviewers critically appraised each individual article using the NOS. Discrepancies between reviewers will be resolved by discussion and by including a third reviewer. The average of two independent reviewer's quality scores will be used to determine whether the articles should be included. Articles with methodological flaws or incomplete reporting of results in the full text will be excluded from the final analysis.

28. * Strategy for data synthesis.

Provide details of the planned synthesis including a rationale for the methods selected. This **must not be generic text** but should be **specific to your review** and describe how the proposed analysis will be applied to your data.

The report of the review will be a narrative summary and will be developed according to the Preferred Reporting Items for Systematic Reviews and Meta-analyses criteria. Information on the studies' characteristics such as publication year; study geographic region, hospitals, design, sample size, percentage of non-adherence to self-care practice in diabetes patients, the age-range of diabetes and response rate will

be extracted from each study using a Microsoft Excel spreadsheet template. Then, data were transferred to Stata (version 14; Stata Corp, College Station, TX) for further analysis. For factors where results will be reported in a similar fashion and had consistent definitions across studies, a meta-analysis will be performed to obtain the associations between these factors and non-adherence to self-care behaviors. The measure of the association from included articles will be adjusted odds ratios (AOR). Standard error and/or 95% confidence interval (CI) of AOR will also be collected. In order to achieve better generalizability of study results, a random-effects model will be applied. The Cochran Q statistic and inverse variance (I^2) statistic will be used to test for heterogeneity. Heterogeneity across studies will be checked="checked" value="1" using the inverse variance (I^2) and Cochran Q statistics and the cutoffs of 25, 50, and 75% will be used to declare the heterogeneity as low, moderate, and severe respectively [29]. In addition, sub-group analysis by types of hospital, study population, and geographical will be considered to estimate the prevalence of non-adherence to self-care practice in the country.

29. * Analysis of subgroups or subsets.

State any planned investigation of ‘subgroups’. Be clear and specific about which type of study or participant will be included in each group or covariate investigated. State the planned analytic approach.

Not applicable

30. * Type and method of review.

Select the type of review and the review method from the lists below. Select the health area(s) of interest for your review.

Type of review

Cost effectiveness
No

Diagnostic
No

Epidemiologic
No

Individual patient data (IPD) meta-analysis
No

Intervention
No

Meta-analysis
Yes

Methodology
No

Narrative synthesis
No

Network meta-analysis
No

Pre-clinical
No

Prevention
No

Prognostic
No

Prospective meta-analysis (PMA)
No

Review of reviews
No

Service delivery
No

Synthesis of qualitative studies
No

Systematic review
Yes

Other
No

Health area of the review

Alcohol/substance misuse/abuse
No

Blood and immune system
No

Cancer
No

Cardiovascular
No

Care of the elderly
No

Child health
No

Complementary therapies
No

COVID-19
No

Crime and justice
No

Dental
No

Digestive system
No

Ear, nose and throat
No

Education
No

Endocrine and metabolic disorders
Yes

Eye disorders
No

General interest
No

Genetics
No

Health inequalities/health equity
No

Infections and infestations
No

International development
No

Mental health and behavioural conditions
No

Musculoskeletal
No

Neurological
No

Nursing
No

Obstetrics and gynaecology
No

Oral health
No

Palliative care
No

Perioperative care
No

Physiotherapy
No

Pregnancy and childbirth
No

Public health (including social determinants of health)
No

Rehabilitation
No

Respiratory disorders
No

Service delivery
No

Skin disorders
No

Social care
No

Surgery
No

Tropical Medicine
No

Urological
No

Wounds, injuries and accidents
No

Violence and abuse
No

31. Language.

Select each language individually to add it to the list below, use the bin icon to remove any added in error.
English

There is not an English language summary

32. * Country.

Select the country in which the review is being carried out from the drop down list. For multi-national collaborations select all the countries involved.

Ethiopia

33. Other registration details.

Give the name of any organisation where the systematic review title or protocol is registered (such as with The Campbell Collaboration, or The Joanna Briggs Institute) together with any unique identification number assigned. (N.B. Registration details for Cochrane protocols will be automatically entered). If extracted data will be stored and made available through a repository such as the Systematic Review Data Repository (SRDR), details and a link should be included here. If none, leave blank.

34. Reference and/or URL for published protocol.

Give the citation and link for the published protocol, if there is one

Give the link to the published protocol.

Alternatively, upload your published protocol to CRD in pdf format. Please note that by doing so you are consenting to the file being made publicly accessible.

No I do not make this file publicly available until the review is complete

Please note that the information required in the PROSPERO registration form must be completed in full even if access to a protocol is given.

35. Dissemination plans.

Give brief details of plans for communicating essential messages from the review to the appropriate audiences.

Do you intend to publish the review on completion?

Yes

36. Keywords.

Give words or phrases that best describe the review. Separate keywords with a semicolon or new line. Keywords will help users find the review in the Register (the words do not appear in the public record but are included in searches). Be as specific and precise as possible. Avoid acronyms and abbreviations unless these are in wide use.

37. Details of any existing review of the same topic by the same authors.

Give details of earlier versions of the systematic review if an update of an existing review is being registered, including full bibliographic reference if possible.

38. * Current review status.

Review status should be updated when the review is completed and when it is published. For newregistrations the review must be Ongoing.
Please provide anticipated publication date

Review_Ongoing

39. Any additional information.

Provide any other information the review team feel is relevant to the registration of the review.

40. Details of final report/publication(s) or preprints if available.

This field should be left empty until details of the completed review are available OR you have a link to a preprint.

Give the link to the published review.
