# Supplementary material for: Non-adherence to self-care and associated factors among diabetes adult population in Ethiopian: A systemic review with meta-analysis
Source: PLoS One. 2021 Feb 10;16(2):e0245862. doi: 10.1371/journal.pone.0245862 (PMC7875372; doi:10.1371/journal.pone.0245862)
Supplement: Attachment — Submitted filename: A Response to Reviewers.docx [file pone.0245862.s007.docx]

**Response to Reviewers**

Response to editor and reviewers’
response to the editor:

We thank you and the reviewers for a thorough reading and constructive criticism of our manuscript and for the opportunity to revise and resubmit. We are pleased to submit the improved research article, including a proposed comment, “**Non-adherence to self-care and associated factors among diabetes adult population in Ethiopian: a systemic review with meta-analysis**”

**EDITOR COMMENT:** the manuscript requires a very extensive revision according to the reviewer's comments, which need to be addressed entirely and properly.

RESPONSE: Thank you for coordinating the review process. We have revised the
manuscript and addressed the reviewer’s comments.

General Comments:

**COMMENT**: Please ensure that your manuscript meets PLOS ONE's style requirements, including those for file naming.

RESPONSE: We have checked and attest that all formatting and style requirements have
been met.

COMMENT: Please change your reference to "p=0.000" to "p<0.001" or as similarly appropriate, as p values cannot equal zero.

RESPONSE: **we changed the p-value from p=0.000 to p<0.001 in the manuscript.**

**COMMENT**: Please change your reference to "p-value=NS to the exact value.

RESPONSE: **we change the reference p-value to the exact value.**

**COMMENT:** Please explain the rationale for the search dates.

RESPONSE: the reason for the search date was limited by 25 February and March 5, 2020, the authors preplanned to search independently and combined the reaching primary article were taken ten days.

**COMMENT**: Please remove any funding-related text from the manuscript.

RESPONSE: we removed the funding statement from the manuscript and write No funding received for this specific study.

**COMMENT:** PLOS requires an ORCID iD for the corresponding author in Editorial Manager on papers submitted after December 6th, 2016. Please ensure that you have an ORCID iD and that it is validated in Editorial Manager.

**RESPONSE: the corresponding author create an ORCID iD.**

**COMMENT:** Please amend either the title on the online submission form (via Edit Submission) or the title in the manuscript so that they are identical.

**RESPONSE: we amend the title so that identical in the system and the manuscript**

**COMMENT:** Please amend the manuscript submission data (via Edit Submission) to include author Emiru Ayalew.

**RESPONSE: we amend by including author Emiru Ayalew in the submission process.**

**COMMENT:** Please amend either the abstract on the online submission form (via Edit Submission) or the abstract in the manuscript so that they are identical.

**RESPONSE: we amend the abstract and make it identical to the online submission and the manuscript.**

**COMMENT:** Please remove your figures from within your manuscript file, leaving only the individual TIFF/EPS image files, uploaded separately.  These will be automatically included in the reviewers’ PDF.

**RESPONSE: the figures are removed within the manuscript and upload separately.**

**COMMENT:** We note that this manuscript is a systematic review or meta-analysis; our author guidelines therefore require that you use PRISMA guidance to help improve reporting quality of this type of study. Please upload copies of the completed PRISMA checklist as Supporting Information with a file name “PRISMA checklist”.

**RESPONSE: we include the PRISMA checklist as supporting information with the file “PRISMA checklist.”**

**RESPONSE TO REVIEWER 1**

REVIEWER COMMENT: In the abstract, among the results authors should include how many articles were included in each analysis.

**RESPONSE: we include a number of primary studies that are included in the analysis.**

REVIEWER COMMENT: The manuscript is not balanced now. In particular, the background paragraph should be shortened.

**RESPONSE: we try to shorten the paragraph of the background**

REVIEWER COMMENT: Authors state that they used a random effect model when Higgin's index was >89.8%. Based on my experience and also considering what authors wrote in their manuscript, this cut-off should be replaced with 50%. Then, authors should repeat the analysis by using a fixed-effect model when I^2 is < or = 50% and a random-effect model when I^2 is >50%.

**RESPONSE: we stated the cut-off point in the heterogeneity had taken low, moderate, and high categories when the I2 values were below 25%, between 25% and 75 %, and above 75%, respectively [39, 40]. Higgin's index I2 >89.8% is not a cut-off point. But, this is our finding of severe heterogeneity, and we have done further analysis**

REVIEWER COMMENT: Authors should include as supplementary material a list of articles which were excluded from the analysis.

**RESPONSE: we uploaded the excluded article as supplementary material.**

REVIEWER COMMENT: Table 1 is not very accurate. Authors should enlist the included studies in alphabetical order or based on the publication date of the article.

**RESPONSE: we enlist the primary study article based on alphabetical order.**

REVIEWER COMMENT: To enrich table 1, authors might include also the prevalence of man / woman in each single study.

**RESPONSE: Most primary articles (66.67%/14/) did not report the prevalence of man/woman non-adherence level of self-care practice. That is why we did not included**

REVIEWER COMMENT: In the manuscript, authors should consider to refer to doi: 10.1016 /j. numecd.2020.03.005.

**RESPONSE: we try to the related concept in this ‘doi’ but, we cannot find the related concept. If you recommend the related concept we are ready to consider it.**
