# Supplementary material for: Non-adherence to self-care and associated factors among diabetes adult population in Ethiopian: A systemic review with meta-analysis
Source: PLoS One. 2021 Feb 10;16(2):e0245862. doi: 10.1371/journal.pone.0245862 (PMC7875372; doi:10.1371/journal.pone.0245862)
Supplement: Attachment — Submitted filename: 3A Response to Reviewers.docx [file pone.0245862.s008.docx]

**Response to Reviewers**

Response to editor and reviewer
we thank you and the reviewers for a thorough reading and constructive criticism of our manuscript and for the opportunity to revise and resubmit. We are pleased to submit the improved research article, including a proposed comment, “**Non-adherence to self-care and associated factors among diabetes adult population in Ethiopian: a systemic review with meta-analysis**”

**RESPONSE TO REVIEWER 1**

REVIEWER COMMENT: Has the statistical analysis been performed appropriately and rigorously? No

**RESPONSE:** we try to explain the statistical analysis appropriately and rigorously on page 8-9, lines 180-205 (including heterogeneity test, publication bias assessment and meta-regression, and sensitivity analysis)

REVIEWER COMMENT: even though the manuscript is significantly improved in comparison with the original version, I still have some concerns about the method of data synthesis used. As a matter of fact, authors should have used a random-effect model when heterogeneity was high and a fixed-effect model when heterogeneity was low, also in the subgroup analyses. If this approach was adopted, it was not clearly declared in the methods.

**RESPONSE:** this is an important comment. We incorporate the means of sub-group analysis the method section on page 9, lines 192-194.

REVIEWER COMMENT: I suggested the authors to discuss their results in the context of the findings published by Cicero et al. (PMID: 32249143), regarding the awareness of diabetes in a well characterized European population. In the rebuttal letter, authors said they could not find the article using the doi (?)... I do not dare to think how they could accurately have done the bibliographic search for their meta-analysis!

**RESPONSE**: we are discuss our results in the context of the findings published by Cicero et al in the discussion part.

REVIEWER COMMENT: finally, the current systematic review and meta-analysis has some deviations from its protocol (as it was registered in PROSPERO). This is a flaw that needs to be addressed.

**RESPONSE**:

That was an editorial problem, we edited the protocol according to the current manuscript.
